# The impact of integrating rabbit haemorrhagic disease virus (K5) release with pindone baiting on wild rabbit populations

**DOI:** 10.1002/ece3.10991

**Published:** 2024-03-11

**Authors:** Kandarp K. Patel, Catherine Austin, Katrina Warner, Marcus Pickett, Aliakbar Khabiri, Mohammadreza Mahzounieh, Farhid Hemmatzadeh, Patrick L. Taggart

**Affiliations:** ^1^ School of Animal and Veterinary Sciences The University of Adelaide Roseworthy South Australia Australia; ^2^ Davies Livestock Research Centre The University of Adelaide Roseworthy South Australia Australia; ^3^ Centre for Invasive Species Solutions Bruce Australian Capital Territory Australia; ^4^ Landscapes Hills and Fleurieu Mount Barker South Australia Australia; ^5^ Marcus Pickett Ecological Services Lobethal South Australia Australia; ^6^ Bush Heritage Australia Victor Harbor South Australia Australia; ^7^ School of Biological, Earth and Environmental Sciences University of New South Wales Sydney New South Wales Australia

**Keywords:** biological control, integrated control, invasive species, *Oryctolagus cuniculus*, pest management, pindone, rabbit biocontrol, rabbit haemorrhagic disease

## Abstract

Several conventional and recently available tools are available for an integrated control of European rabbits in Australia. We quantified the impact of the release of rabbit haemorrhagic disease virus K5 (RHDV K5, hereafter K5) and pindone (2‐pivalyl‐1,3‐indandione) baiting at 13 sites within Cudlee Creek fire scar in the Adelaide Hills, South Australia. K5 release was followed by pindone baiting between December 2021 and March 2022; the application of both control methods followed industry best practice. We counted rabbits using spotlights before and after the application of both control methods. Fly samples and livers from dead rabbits were collected to track K5 transmission within and between sites, and to detect the natural circulation of rabbit haemorrhagic disease virus 2 (RHDV2). K5 release had minimal impact on rabbit populations, with treated populations increasing by a mean of 65.5% at 14 days post‐release and 27.9% at 77 days post‐K5 release across all sites, comparable to the changes at control sites. K5 detection in flies up to 77 days post its release, and its detection in rabbit livers, demonstrates that it can survive and transmit in the environment for prolonged periods and that it can lethally infect some rabbits. This limited impact of K5 is consistent with previous studies and may be explained by pre‐existing RHDV/RHDV2 immunity in the target populations or the presence of young rabbits with natural innate RHDV immunity. The detection of K5 in flies from control sites demonstrates that it was vectored beyond its release location. A reduction in rabbit counts post‐pindone baiting was observed at most treatment sites, with a mean population reduction of 36.6% across all sites. Landholders need to carefully and strategically plan their integrated rabbit control programmes. Not all combinations of controls, even if theoretically logical, achieve meaningful outcomes for rabbit management.

## Graphical abstract

This study quantified the impact of the release of rabbit haemorrhagic disease virus K5 (RHDV K5, K5 hereafter) and pindone baiting at 13 sites within Cudlee Creek fire scar in the Adelaide Hills region, South Australia. K5 release had minimal impact on rabbit populations, whereas a reduction in rabbit counts post‐pindone baiting was observed at most treatment sites. Landholders need to carefully and strategically plan their integrated rabbit control programmes as not all combinations of controls, even if theoretically logical, achieve meaningful outcomes for rabbit management.

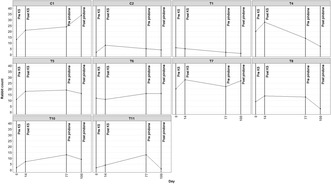

## 1 INTRODUCTION

The European rabbit is one of the most important vertebrate pest species in Australia impacting 322 species listed as threatened under the Environmental Protection Biodiversity Conservation Act (Kearney et al., 2019). Rabbits additionally impact on the Australian agricultural industries, with economic costs estimated at >200MAUD annually (Gong et al., 2009). Over many decades, landholders, local and the state/territory governments have invested substantial labour and financial resources in rabbit management. Rabbit management was solely achieved using physical and chemical methods before the availability of biocontrol tools. Physical methods include destroying warrens/aboveground harbour, trapping or shooting rabbits; and chemical methods include poison baiting with sodium monofluroacetate or pindone (2‐pivalyl‐1,3‐indandione), and chloropicrin pressure or phosphine diffusion fumigation.

Several studies have demonstrated the cost‐efficiency and the effectiveness of rabbit control programmes using pindone poisoning and other physical control methods. For example, Cooke and Hunt (1987) showed that the poisoning followed by warren destruction by ripping was more beneficial, but less cost‐effective than individual methods, in semi‐arid hilly country in South Australia. Similarly, Cooke (1981) demonstrated that a pindone poisoning was highly cost‐effective for managing roadside rabbit warrens. Williams and Moore (1995) reported that pindone poisoning followed by warren destruction by ripping improved effectiveness and cost compared to other combinations using chloropicrin pressure or phosphine diffusion fumigation.

However, the introduction of rabbit biocontrols to Australia significantly changed the control and management of rabbits. In 1950, myxoma virus was released as Australia's first rabbit biocontrol. Following this, rabbit numbers declined by up to 90%–99% in some areas. However, rabbit populations subsequently recovered to some extent, possibly due to rabbits developing genetic resistance to infection and the virulence of the virus declining (Fenner et al., 1953; Marshall & Fenner, 1960; Ratcliffe et al., 1952). A second biocontrol virus, rabbit haemorrhagic disease virus (RHDV, strain CZECH, GI.1 CAPM 351; hereafter CZECH), was brought into Australia in the early 1990s. Experiments with CZECH were undertaken on Wardang Island, South Australia, to assess its effectiveness for rabbit control. Subsequently, the virus escaped during trials in 1995 and reached mainland South Australia where it decimated rabbit populations (Cooke, 1996; Mutze et al., 1998). However, as the virus became established, rabbit populations started to develop immunity to CZECH, with some populations also developing a degree of genetic resistance (Elsworth et al., 2012).

The recovery of rabbit populations following the introduction of CZECH again led scientists in search of additional biocontrol options. RHDV, K5 (GI.1a also known as 08Q712, hereafter K5) was subsequently selected for release, with the intention that it would complement or ‘boost’ RHDV‐mediated rabbit biocontrol. K5 was able to overcome partial immunological cross protection from previous infection with the benign rabbit calicivirus (RCV‐A1) (Cooke et al., 2018), and was able to better infect wild rabbits considered to be more genetically resistant. Consequently, K5 was then released nationally in 2017 with the intention that it would act as a landscape‐scale rabbit biocontrol in Australia (Cox et al., 2019; Mutze et al., 2014).

K5 can also be accessed and released locally by landholders to assist with targeted rabbit management. However, previous studies have suggested that, in isolation, its benefit for rabbit management may be limited (Cox et al., 2019). This is problematic as anecdotal evidence suggests that landholders commonly lose interest in rabbit control if they do not achieve their desired population reduction. Further to this, the release of K5 is commonly not integrated with any other form of rabbit control and is commonly perceived to be a ‘silver bullet’ control method; these releases also often occur at the wrong time of year when benefits would be expected to be limited (Taggart et al., 2022, 2024). Despite this, other similar biocontrols, such as CZECH and RHDV2 circulate naturally in Australia at landscape scales and have made phenomenal contributions to rabbit management (Mahar et al., 2018).

In contrast to the natural circulation of some biocontrols, the application of conventional control methods and their contribution to reduced rabbit populations does not occur in the absence of proactive landholders. The application of conventional rabbit control methods also does not stop the spread of RHDV (Mutze et al., 2010), and hence to achieve maximum rabbit population reduction land managers should always integrate the application of biocontrols, such as K5, with conventional methods where possible.

In Dec 2019, the ‘Cudlee Creek’ bushfire burnt approximately 23,000 ha of land in the mixed peri‐urban and rural Adelaide Hills region of the Mount Lofty Ranges, extending from the Cudlee Creek area southeast and east to the Brukunga, Harrogate and Mount Torrens areas (Government of South Australia 2020). To manage the amplified impacts of rabbits following the bushfires, Landscapes Hills and Fleurieu, a regional Government of South Australia agency, ran rabbit bait distribution days to provide landholders with K5 and pindone‐treated carrots. This initiative was part of a region‐wide rabbit control programme.

The aim of this study was to investigate the benefit of integrating K5 release with pindone baiting for the control of rabbit populations. Accordingly, we investigated the impact of the release of K5 followed by the impact of the pindone baiting on rabbit populations at 13 sites within the 2019 Cudlee Creek fire scar.

## 2 MATERIALS AND METHODS

### 2.1 Study sites

All study sites were located within the 2019 Cudlee Creek fire scar, <1‐h drive from Adelaide, South Australia. This study used a sub‐sample of properties participating in a larger rabbit control programme being run by Landscapes Hills and Fleurieu. Landholders participating in this programme were contacted to participate in our study. In total, 13 landholders participated in our study (Supplementary Material S1). Initially, 11 sites were allocated to the treatment group (K5 release followed by pindone baiting) and two sites were allocated to the control group (no K5 release and no pindone baiting). Treatment and control sites were a minimum of 1.6 km (mean = 7 km; max = 14.5 km) apart, whereas the mean distance between treatment sites was 7.7 km (range: 1.2–20.8 km). The closest non‐participating site with K5 release was 0.5 and 1.5 km from control sites C1 and C2 respectively. Transects and/or search areas were identified within each site based on hotspots of rabbit activity.

### 2.2 K5 release and pindone baiting

Free‐feeding with carrots was undertaken for three consecutive nights at all sites prior to K5 release. On the fourth consecutive night, following free‐feeding, 1 kg of K5‐treated or ‐untreated chopped carrots were distributed to participating landholders at treatment and control sites respectively. Landholders were given instructions that they were to broadcast the carrot bait at the same free‐feed locations on the night they received the bait (8 December 2021). The preparation of all carrot bait was conducted by Landscapes Hills and Fleurieu in accordance with the manufacturers' instructions All K5 used in carrot baits was produced by and accessed through the only commercial RHDV laboratory in Australia (New South Wales Department of Primary Industries).

Similarly, free‐feeding with carrots was undertaken three times at all sites prior to pindone baiting, on the nights of the 14th, 17th and 20th of February 2022. Following free‐feeding, 3 kg of pindone‐treated or ‐untreated chopped carrots were distributed to all participating landholders on 23 February 2022, with instructions that they were to be broadcast at the free‐feed locations on the nights of the 23rd February, 26th February and 1st March. All pindone‐treated carrots were prepared in accordance with the standard operating procedure for pindone baiting by Landscapes Hills and Fleurieu (https://pestsmart.org.au/wp‐content/uploads/sites/3/2021/03/RAB004‐SOP.pdf).

### 2.3 Rabbit counts

Treatment and control sites were located based on rabbit hotspots identified at candidate properties. Rabbits were counted during hours of darkness (between end and beginning of nautical twilight, but generally concluding well before the latter) using an area‐search technique. Area‐search surveys involved a walked ramble survey (consistent start point) of up to 10 min duration and using a combination of thermal monocular (principally) and white‐light spotlight (supplementary) to detect rabbits within the search area boundary. Due to unavoidable heterogeneity in site configuration (candidate properties were constrained), survey area varied across sites (mean ~ 1 ha), however, survey method and coverage within a given site was equivalent across surveys.

Rabbit counts were conducted on three consecutive nights leading up to the K5 release and pindone baiting, and again on three consecutive nights 14 days after K5 release and 23 days after pindone baiting to estimate the change in rabbit numbers. The maximum of the three consecutive rabbit spotlight counts was used to estimate the rabbit population on each occasion (prior to K5 release, post‐K5 release, prior to pindone baiting, post‐pindone baiting).

### 2.4 Fly and carcass sampling

To detect K5 activity in flies, commercial fly traps (Envirosafe Fly Trap, Australia) were deployed adjacent to rabbit hotspots at survey sites. Flies were sampled 1 day before K5 release and on the 14th and 31st days after K5 release. One pre‐K5 fly trap was not installed and one post‐K5 fly trap was destroyed by livestock. Fly traps were also set on the night of pindone baiting and were collected 23 days later.

Landholders were encouraged to actively search their study sites during K5 release/pindone baiting period for dead rabbit carcasses. Two rabbit carcasses were found at one study site, and another carcass was found at a second site between 12 and 14 days after K5 release. One rabbit carcass was found at a third site 23 days after pindone baiting. Liver or leg bone was collected from all carcasses for RHDV molecular testing.

All fly, liver and leg bone samples were frozen at −20°C until molecular testing was conducted at the University of Adelaide virology laboratory (Roseworthy, SA).

### 2.5 RNA extraction and cDNA synthesis

Viral RNA from liver and fly samples was extracted using the QIAamp Viral RNA kit (Qiagen, Hilden, Germany) according to the manufacturer's instructions. The extracted RNA was quantified using a NanoDrop One Microvolume UV Spectrophotometer (Thermo Fisher Scientific, Waltham, USA). cDNA was synthesised using ~1 μg of RNA using random hexamer and gene‐specific primers using SuperScript™ IV Reverse Transcriptase (Thermo Fisher Scientific, Waltham, USA).

### 2.6 RT‐qPCR and high‐resolution melting analysis (HRM) using RHDV universal primers

The primer set F2‐R3 was designed using multiple alignment of polyprotein gene of RHDV in ClustalW programme. The primers with the closest match to all Australian RHDV strains were selected for synthesis. The selected primers were used to run the RT‐qPCR for the detection of RHDV (Czech and RHDV‐K5) and RHDV2 (Table 1). To detect the rabbit housekeeping gene two primers (Rabbit HK F and R) were selected to amplify a 110 bp block of 12S ribosomal RNA from *Oryctolagus cuniculus* mitochondrion (Accession number MN518689.1).

**TABLE 1:** Primers used for detecting RHDV and RHDV2 in RT‐qPCR and high‐resolution melting analysis.

Primer name	Sequence	PCR product size (bp)
F2‐MM‐Calici‐UniRHDV	GCCCAGCCAGCGTACAT	372
R3‐MM‐Calici‐UniRHDV	TCAGACATAAGAAAAGCCATTGG	
F‐MM‐Rabit HK‐110	CAAAAGTAAGCTCAATTACCACCGTA	110
R‐MM‐Rabit HK‐110	ATAAGGGCTTTCGTATATTCGGAA	

The reaction mixture was prepared using Type‐it HRM PCR Kit (Qiagen, Hilden, Germany). DNA amplification was conducted in a 48 well microplate (Illumina, San Diego, CA, USA). Each well contained 10 μL reaction solution, including 5 μL HRM SuperMix, 1 μL DNA template (approximately 15 ng), 1 μL of each primer (0.2 nmol) and 2 μL nuclease‐free water. The reaction was conducted using an Illumina Thermal Cycler with preheating activation for 2 min followed by 40 PCR cycles of three steps: denaturation at 95°C for 15 s, annealing at 58°C for 45 s then extension at 72°C for 15 s. HRM was performed at 55–95°C at the rate of 0.1°C. Results were analysed via EcoStudy software (version 5.0, Illumina, San Diego, CA, USA). To check the accuracy of the test, the PCR products were analysed with electrophoresis in 1.5% agarose gel and staining with Gel Red to confirm the accuracy of PCR product bands. The same PCR conditions were used for the genotype‐specific and rabbit housekeeping genes.

The same primer set was used for HRM and q‐PCR. The reaction mixture was prepared using Type‐it HRM PCR Kit (Qiagen, Hilden, Germany). DNA amplification was conducted in a 48 well microplate (Illumina, San Diego, CA, USA). Each well contained 10 μL reaction solution, containing 5 μL HRM SuperMix, 1 μL DNA template (approximately 15 ng), 1 μL of each primer (0.2 nmol) and 2 μL nuclease‐free water. The reaction was conducted using an Illumina Thermal Cycler with preheating activation for 2 min followed by 40 PCR cycles of three steps: denaturation at 95°C for 15 s, annealing at 58°C for 45 s then extension at 72°C for 15 s. HRM was performed at 55–95°C at the rate of 0.1°C. The results were analysed via EcoStudy software (version 5.0, Illumina).

The standard curves for both K5 and GI.2 strains were created using EcoStudy software (version 5.0, Illumina, San Diego, CA, USA). To calculate the absolute copy number of the viral genome in spike experiment, a standard curve was created for each PCR product. The limit of detection of the RT‐qPCR test per 1 μL of sample (representing 10 mg of tissue) for K5 strain was 3.89 and for GI‐2 strain was 2.59, when the purified PCR product was used to make serial dilution. The HRM analysis showed two separate melting curves when RHDV GI and GII were used in the test. The HRM profile for GI and GII generated one melting peak each at 86.4 and 87.7°C respectively. The results for HRM were confirmed in sequencing with 100% identity to the reference strains for each genotype.

### 2.7 Sequencing

To confirm the accuracy of the HRM analysis, the PCR product of F2 and R3 primers were purified and submitted for sequencing in both forward and reverse directions. Each PCR product was submitted to Australian Genome Research Facility Ltd (AGRF) for Sanger sequencing. The sequences were edited using BioEdit (v.7.0.4.1) software and Blasted against the available reference strains in GenBank.

### 2.8 Statistical analysis

All descriptive analyses were performed in R statistical software (v 4.1.1; R Core Team, 2020). Student's *t*‐test was used to assess changes in maximum rabbit count between and within each treatment method. Three sites (T2, T3 and T9) were excluded from the rabbit count analysis following very low rabbit counts (<2) during the pre‐K5 release spotlight count survey but were included in the sites used for fly testing data analysis.

## 3 RESULTS

### 3.1 Impact of K5 release

**FIGURE 1:**
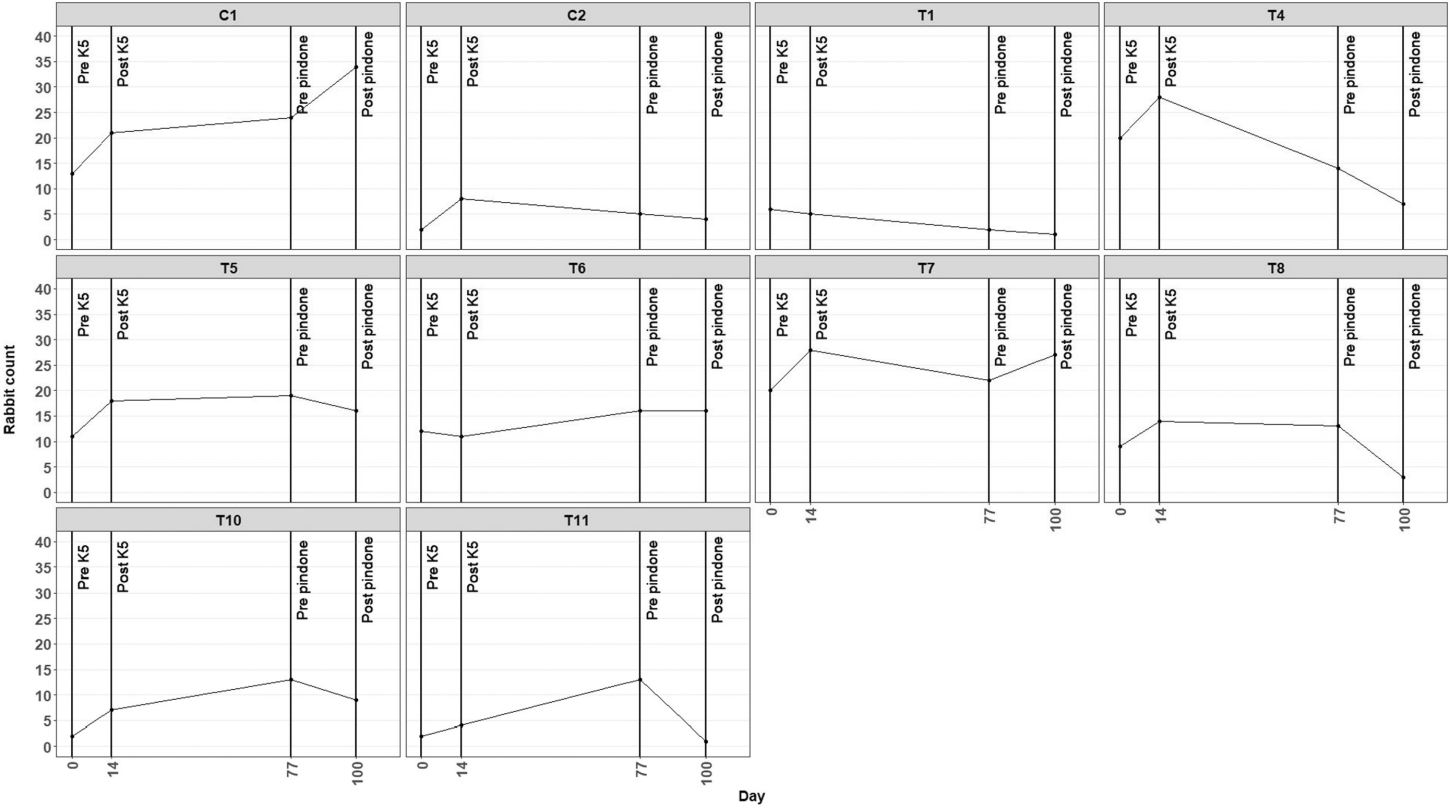
Rabbit counts at control (C1, C2) and treatment (T1, T4, T5, T6, T7, T8, T10, T11) sites before (line labelled ‘Pre‐K5’) and after (line labelled ‘Post‐K5’) application of K5 and before (line labelled ‘Pre‐pindone’) and after (line labelled ‘Post‐pindone’) application of pindone baiting. All study sites were located within the 2019 Cudlee Creek fire scar.

Fourteen days post‐K5 release, rabbit populations had significantly (*p* = .02) increased by a mean of 66% (range: 16.7% decrease to 250% increase) across all treatment sites (Figure 1). A reduction in the local rabbit population was only observed at T1 (16.7% reduction) and T6 (8.3% reduction) 14 days post‐K5 release; rabbit populations increased at the six other treatment sites where counts occurred over this same period. Evidence of K5 transmission via flies was detected at five of the total 11 treatment sites post‐K5 release. This suggested that at least some rabbits had been killed at these five sites, or neighbouring sites, consistent with three K5‐positive liver samples collected 12–14 days after release from two of these five sites (T2 and T7). However, none of the five sites where K5 was detected in flies showed a reduction in the maximum rabbit count 14 days after K5 release. No evidence of K5 transmission via flies was observed at either treatment site where reductions in rabbit populations were observed 14 days post‐release. Rabbit populations had increased by 61.5% and 300.0% at C1 and C2 control sites at 14 days post‐K5 release respectively. K5 was not detected in flies at either control site at this time point, although RHDV2 was detected in flies 14 days post‐K5 release. The mean increase in the rabbit populations at control sites was not significantly higher than the increase in the treatment sites (*p* = .18).

From 14 to 77 days post‐K5 release (77 days post‐K5 release = immediately prior to pindone baiting) rabbit populations increased by a mean of 28% (range: 60% decrease to 225% increase) across all treatment sites (Figure 1). However, this change was not significant (*p* = .89). A reduction in the rabbit population was observed at T1 (60% reduction), T4 (50% reduction), T7 (21% reduction) and T8 (7% reduction); rabbit populations increased at the four other treatment sites where counts occurred over this same period. Evidence of K5 transmission via flies was detected at two of the 11 treatment sites at 31 days post‐K5 release and at all the 11 treatment sites, and both control sites, 77 days post‐K5 release (prior to pindone baiting; Table 2). This included the detection of K5 in flies at all four sites where there was evidence of rabbit population reduction between 14 and 77 days post‐K5 release, and at all four sites where there was no evidence of rabbit population reduction during this same period. Again, this suggested that at least some rabbits had been killed at these sites, or neighbouring sites, post‐K5 release. Rabbit populations increased at one control site and decreased at the other between 14 and 77 days post‐K5 release, and K5 was detected at both control sites 77 days post‐release, but not at 31 days post‐release. RHDV2 was also detected at one control site at 31 days post‐release and at both sites at 77 days post‐release, suggesting that it may have also contributed to changes in rabbit populations at these sites.

**TABLE 2:** Genotyping results, following RT‐qPCR/HRM analysis, in fly samples collected at control (C1, C2) and treatment (T1, T2, T3, T4, T5, T6, T7, T8, T9, T10, T11) sites before and after the application of K5 release or pindone baiting.

Site	Rabbit count	Fly samples collected at			
		Pre‐K5 release	14 days post‐K5 release	31 days post‐K5 release	77 days post‐K5 release (pre‐pindone baiting)
C1	Y	NEG	RHDV2	RHDV2	K5 & RHDV2
C2	Y	NEG	RHDV2	NEG	K5 & RHDV2
T1	Y	NEG	NEG	NEG	K5
T2	N	Trap not installed	NEG	NEG	K5 & RHDV2
T3	N	No sample	K5	NEG	K5
T4	Y	NEG	K5	NEG	K5
T5	Y	NEG	NEG	K5	K5
T6	Y	No sample	NEG	NEG	K5
T7	Y	No sample	K5	NEG	K5
T8	Y	No sample	NEG	NEG	K5
T9	N	NEG	K5 & RHDV2	NEG	K5
T10	Y	NEG	K5	K5	K5
T11	Y	NEG	NEG	K5	K5

*Note*: All study sites were within the 2019 Cudlee Creek fire scare. No rabbit counts were conducted on three treatment sites (T2, T3, T7) following very low rabbit counts pre‐K5 release. Only RT‐qPCR/HRM‐positive samples were sent for genotyping analysis.

No fly trap was installed at one treatment site (T2) and no flies were trapped at four treatment sites (T3, T6, T7, T8) prior to K5 release. All fly samples collected prior to K5 release tested negative to both RHDV (Czech and K5) and RHDV2 (Table 2). K5 was also detected in flies from one (T3) of 3 treatment sites where no post‐K5 release rabbit counts were conducted (Table 2).

### 3.2 Impact from pindone baiting

Twenty‐three days post‐pindone baiting, rabbit populations had decreased by a mean of 36.6% (range: −92.3% decrease to 22.7% increase) across all treatment sites (Figure 1). This change in rabbit numbers was marginally not significant (*p* = .08). A reduction in the rabbit population was observed at T1 (50% reduction), T4 (50% reduction), T5 (15.8% reduction), T8 (76.9% reduction), T10 (30.8% reduction) and T11 (92.3% reduction) 23 days post‐pindone baiting; rabbit populations increased at one treatment site and were unchanged at another where counts occurred over this same period. Rabbit populations increased by 41.7% at one control site and decreased by 20% at the other control site 23 days post‐pindone baiting. The mean increase in the rabbit populations at control sites was not significantly higher than the increase in the treatment sites (*p* = .16).

Evidence of K5 transmission via flies prior to pindone baiting (77 days post‐K5 release) was evident by detection of K5 at all treatment sites including those (T2, T3 and T9) with no rabbit counts (Table 2). Transmission of K5 via flies from treatment to control sites was evident from the detection of K5 in fly samples at control sites. A single liver sample collected from T4 at 14 days post‐pindone baiting tested positive to K5. RHDV2 was also detected in flies from T2 and both control sites.

### 3.3 Combined impact from K5 release and pindone baiting

An overall reduction in rabbit population was observed at T1 (83.3% reduction), T4 (65.0% reduction), T8 (66.7% reduction) and T11 (50% reduction). However, rabbit populations increased at four treatment sites: T5 (45.5% increase), T6 (33.3% increase), T7 (35.0% increase) and T10 (350.0% increase). Overall, 100 days after the application of both control tools, the rabbit population across all treatment sites had increased by 25% (range: −83.3% decrease to 350.0% increase). This change in rabbit number was not significant (*p* = .78). Rabbit population reduction post both treatments, K5 release and pindone baiting, was observed at only one (T1) of 8 (12.5%) treatment sites whereas an increase post both treatments was observed at one treatment site (T7). Across the entire study period, the rabbit population increased by 161.5% at one control site (C1) and 100% at the other control site (C2).

## 4 DISCUSSION

This study assessed the impact of K5 release, pindone baiting and their successive application on rabbit populations. The findings from this study show that the application of K5 in early summer achieves only limited rabbit population control, but that moderate population control (mean 37% reduction) can be achieved using pindone baiting in late summer/early autumn. To our knowledge, this is the first study to detect K5 in flies post its release, and one of few (Jenckel et al., 2022; Peng et al., 2023) to demonstrate that its transmission can continue for prolonged periods (minimum 77 days post‐release), or at least that it remained detectable in flies for this period.

### 4.1 Impact from K5 release

The impact of K5 observed in this study was consistent with findings reported by Cox et al. (2019), who report that on average K5 release did not reduce rabbit abundance across 22 professionally monitored sites in Australia. These limited impacts on rabbit populations from K5 release may be explained by several reasons:

- Neither study (this study, nor Cox et al., 2019) undertook RHDV serology within the target rabbit populations prior to K5 release. Rabbits at K5 release sites may have had some existing immunity to K5 due to previously having been exposed to other RHDV variants (inc. RHDV2) (Patel et al., 2022). The detection of RHDV2 in flies at both control sites at the time of K5 release suggests that rabbits at control, and potentially treatment, sites may have recently been exposed to RHDV2. Similar observations have also been reported in laboratory rabbits (Calvete et al., 2018).
- This study was conducted in early summer under the assumption that the local rabbit population would comprise few young rabbits less than 10 weeks of age (Taggart et al., 2022). However, in Australia, 2021 was a La Nina year, resulting in much of the country experiencing higher than average annual rainfall and rainfall at unusual times of the year. This would be expected to facilitate an extended rabbit breeding season, and indeed several landholders observed an abundance of young rabbits at the K5 release sites post its release. Landholder observations were also supported by an increase in rabbit counts at some sites post‐K5 release. Young rabbits, less than 10 weeks of age, have innate immunity to lethal RHDV infections and become immune to RHD for life if exposed at this young age (Neave et al., 2018).

Evidence of K5 in flies at some treatment sites 14 and 31 days post‐K5 release suggests that the virus lethally infected some rabbits post‐release, resulting in subsequent transmission to other sites via flies after their interaction with K5‐infected rabbit carcasses.

### 4.2 Impact from pindone baiting

The impact of pindone baiting on rabbit populations observed in this study was consistent with previous trials (Oliver et al., 1982; Oliver & Wheeler, 1978). Pindone is highly lethal to rabbits of all ages. However, the increase in the maximum rabbit count at one treatment site could be attributed to a low pindone concentration in carrot distributed at the site, or low uptake of the pindone carrots at the site due to the rabbits being particularly neophobic/bait‐shy or social dominance hierarchies where socially dominant rabbits prevent sub‐ordinate individuals from accessing food resources (Iannella, 2018). The mortality achieved in rabbit populations through pindone baiting has been demonstrated to be dose‐dependent, with a lower amount of pindone on individual carrot pieces resulting in lower population reduction (Oliver & Wheeler, 1978). Carrots in this study were treated with pindone by the supplier and hence minimal variation in the pindone concentration on the carrots was expected. However, deployment of the pindone‐treated carrots was conducted by landholders, and it is possible that not all landholders distributed this treated carrot as directed to do so. Within‐site variation in the distribution of pindone‐treated carrots by landholders may have led to sub‐optimal levels of pindone concentration in rabbits leading to sub‐lethal concentrations of pindone in rabbits. However, there could be other unobserved and/or unmeasured reasons associated with the increase in rabbit counts at T7.

### 4.3 K5 transmission by flies

Evidence of K5 in flies at both control sites demonstrates that it is capable of being naturally transmitted via flies from release points, with transmission occurring over a minimum of 1.6 km and potentially up 14.5 km from the treatment sites included in the study. However, it could have been possible that the K5 detection on the control sites may be due to the K5 transmission form other non‐participating sites with K5 releases. This finding is consistent with the other reports of field transmission of RHDV by flies (Asgari et al., 1998; McColl et al., 2002), and evidence of its localised, natural circulation in Western Australia (Jenckel et al., 2022; Peng et al., 2023).

Evidence of K5 in fly samples prior to pindone baiting at all treatment sites additionally demonstrates that it is capable of persisting in the environment for multiple months (77 days between K5 release and initial pindone baiting), presumably due to it causing ongoing infection/mortality in rabbit populations. It is therefore possible that some reduction in rabbit numbers post‐pindone baiting may be attributed to lethal K5 infections. This is supported by the recovery of a K5‐positive carcass post‐pindone baiting. However, as there was no significant reduction in rabbit counts 77 days post‐K5 release, it seems unlikely that it was responsible for much of the observed reductions in rabbit populations post‐pindone baiting.

### 4.4 Combined impact from K5 release and pindone baiting

Though the overall impact from K5 release and pindone baiting was lower than expected, the current rabbit management programme was able to achieve higher reduction in the rabbit populations at treatment sites compared to the control sites. This suggested that the integration of K5 release and pindone baiting may be able to restrict increase in rabbit populations to some degree otherwise expected where no rabbit management is undertaken. Landholders can lose trust and interest in applying any subsequent integrated rabbit controls if the initial control activity do not achieve the expected reduction in rabbit population. The findings from this study reiterates the importance of the ongoing rabbit control post application of a rabbit control activity.

### 4.5 Impact and timing of K5 release and pindone baiting

Pindone baiting can be undertaken any time of the year as, unlike K5, its effectiveness is not influenced by rabbit age class. However, it is recommended that pindone baiting, and any rabbit control is undertaken during summer and autumn when there is little green grass available to support breeding and their population is at a low point in its annual fluctuating cycle. Scarce food resources and higher temperatures at this time of year additionally reduce the chances that rabbits will survive if their populations are subject to disruptive controls, such as warren ripping or aboveground harbour destruction. K5 release should typically be undertaken in late summer or early autumn when there are few young rabbits present in the population that may possibly be immunised from an untimely virus release. However, more research needs to be done into the timing of release of K5 pre‐ or post‐application of any conventional rabbit control method due to the temporally varied composition of rabbit populations, in terms of young and adult rabbits, across different regions in Australia.

### 4.6 Implications for the future rabbit management programmes

Pindone baiting achieved a greater average reduction in rabbit populations compared to K5 release. However, the integration and sequencing of both control methods achieved only inadequate rabbit population reduction. Due to their high maximum annual population growth rate (Caley & Morley, 2002) effective rabbit management is said to require ≥95% population reduction (Williams et al., 1995); in this study, the integration and sequencing of K5 release and pindone baiting did not achieve such high levels of rabbit population reduction at any sites. This highlights the need for a well‐planned, strategic and integrated approaches to rabbit control. Positive benefits of integrating multiple conventional rabbit control methods have been demonstrated by Williams and Moore (1995), and the benefits of integrating biocontrols with conventional control methods have been reported by Mutze et al. (2010). The sub‐optimal success in rabbit control in this study highlights that future rabbit management programmes should apply integration approaches applied by the previous studies yielding positive benefits to formulate a successful integration of the current biocontrol and the conventional tools. This study also shows that though individual control options may lead to higher rabbit knockdown than other individual tools, the planning and strategic integration and application of tools may be needed to achieve maximum rabbit control.

### 4.7 Study limitations

Rabbit counts at all sites did not use equivalent methodology due to limitations from, for example, infrastructure, terrain and vegetation cover at some sites. However, methodology was always kept the same within a site; for example, pre‐K5 release, post‐K5 release, pre‐pindone baiting and post‐pindone baiting rabbit counts were always conducted in the same manner at any particular site. This enabled us to robustly quantify changes in rabbit populations within sites but may limit the degree to which comparisons can be made across sites.

## 5 CONCLUSION

This study shows that, although K5 is likely to be naturally transmitted via flies, its release in late spring or early summer is unlikely to substantially reduce rabbit populations in the Adelaide Hills. This highlights that, despite the best protocols and advice, local breeding conditions need to be assessed prior to releasing K5. In contrast, pindone baiting in late summer/early autumn resulted in an average population reduction of 36.6%, which may be leveraged through effective integration with another rabbit control method.

## AUTHOR CONTRIBUTIONS

**Kandarp K. Patel:** Conceptualization (equal); data curation (equal); formal analysis (equal); investigation (equal); methodology (equal); resources (equal); software (equal); supervision (equal); visualization (equal); writing – original draft (equal); writing – review and editing (equal). **Catherine Austin:** Data curation (equal); funding acquisition (equal); project administration (equal); resources (equal); writing – review and editing (equal). **Katrina Warner:** Data curation (equal); funding acquisition (equal); project administration (equal); resources (equal); writing – review and editing (equal). **Marcus Pickett:** Data curation (equal); project administration (equal); writing – review and editing (equal). **Aliakbar Khabiri:** Investigation (equal); methodology (equal); writing – review and editing (equal). **Mohammadreza Mahzounieh:** Investigation (equal); methodology (equal); writing – review and editing (equal). **Farhid Hemmatzadeh:** Data curation (equal); investigation (equal); methodology (equal); writing – review and editing (equal). **Patrick L. Taggart:** Data curation (equal); formal analysis (equal); investigation (equal); methodology (equal); validation (equal); visualization (equal); writing – original draft (equal); writing – review and editing (equal).

## FUNDING INFORMATION

The funding for this study was jointly provided by the Hills and Fleurieu Landscape Board (Department of Environment and Water, Government of South Australia) under the Local Economic Recovery project in partnership with the Department of Primary Industries and Regions, Government of South Australia and Australian Government through the Australian National Disaster Recovery Funding Arrangements.

## CONFLICT OF INTEREST STATEMENT

Co‐authors, CA and KW, were employed by one of the funding agencies while undertaking this study. CA and KW did not have any role in the design of the study or data analysis.

## Supporting information


Data S1

Appendix S1

## Data Availability

The data that support the findings of this study are openly available in Figshare at https://doi.org/10.25909/24796977.
