# Supplementary material for: The impact of integrating rabbit haemorrhagic disease virus (K5) release with pindone baiting on wild rabbit populations
Source: Ecol Evol. 2024 Mar 11;14(3):e10991. doi: 10.1002/ece3.10991 (PMC10928239; doi:10.1002/ece3.10991)
Supplement: Supplementary file 2 — Appendix S1 [file ECE3-14-e10991-s002.docx]

## Supplementary materials

Supplementary material 1


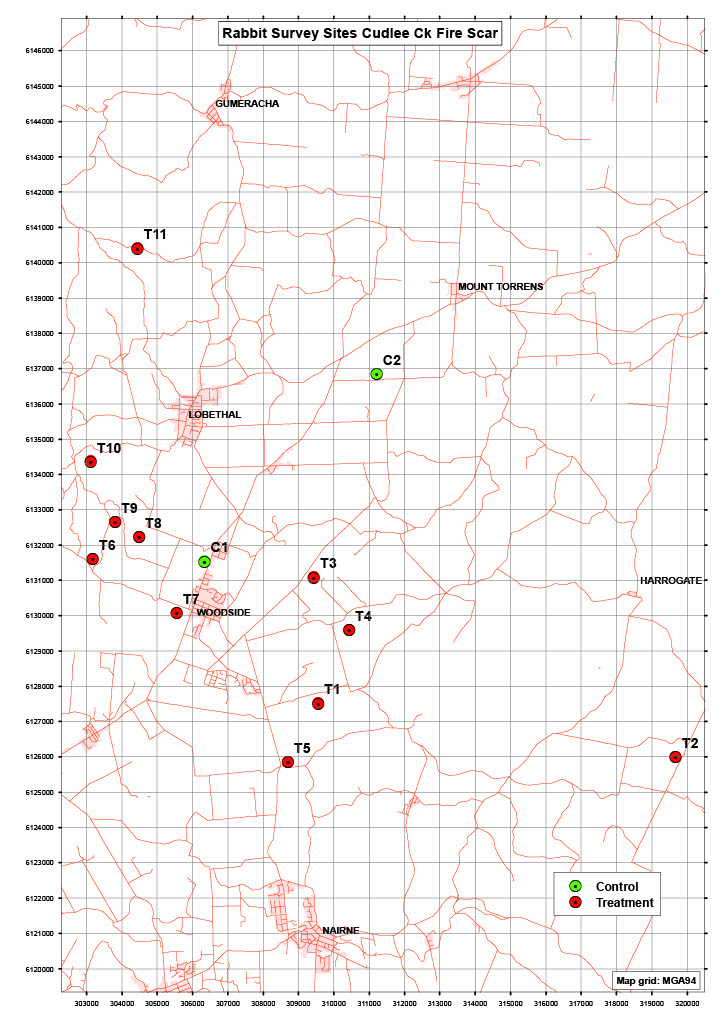


Figure 1. Study site locations for control (C1, C2) and treatment (T1, T2, T3, T4, T5, T6, T7, T8, T9, T10, T11) sites. All sites are contained within the 2019 Cudlee Creek fire scar, South Australia. Map uses MGA94 easting and northing coordinate system.
